# Health anxiety and related factors among pregnant women during the COVID-19 pandemic: a cross-sectional study from Iran

**DOI:** 10.1186/s12888-021-03092-7

**Published:** 2021-02-15

**Authors:** Najmieh Saadati, Poorandokht Afshari, Hatam Boostani, Maryam Beheshtinasab, Parvin Abedi, Elham Maraghi

**Affiliations:** 1 Obstetrics and Gynecology, Fertility Infertility and Perinatology Research Center, Ahvaz Jundishapur University of Medical Sciences, Ahvaz, Iran; 2 Midwifery Department, Reproductive Health Promotion Research Center, Ahvaz Jundishapur University of Medical Sciences, Ahvaz, Iran; 3 Department of Psychiatry, Ahvaz Jundishapur University of Medical Sciences, Ahvaz, Iran; 4 Department of Biostatistics and Epidemiology, Faculty of Public Health, Ahvaz Jundishapur University of Medical Sciences, Ahvaz, Iran

**Keywords:** COVID-19, Corona virus, Health anxiety, Pregnant women

## Abstract

**Background:**

The COVID-19 pandemic has affected many countries around the world and Iran was no exception. The aim of this study was to evaluate health anxiety of Iranian pregnant women during the COVID-19 pandemic.

**Methods:**

In this cross-sectional study, 300 pregnant women in different trimesters (*n* = 100 in each trimester) were recruited. A demographic questionnaire and the Health Anxiety Questionnaire were used to collect data. Scores of < 27, 27–34 and more than 35 were defined as low, moderate and high health anxiety, respectively. Due to nationwide restrictions, data were collected through social media groups. Chi-square tests, ANOVA and multiple linear regression were used to analyze the data.

**Results:**

Mean (SD) total anxiety scores were 22.3 ± 9.5, 24.6 ± 9.3 and 25.4 ± 10.6 in the first, second and third trimesters of pregnancy, respectively. 9, 13 and 21% of women had severe anxiety in the first, second and third trimesters of pregnancy, respectively. Women in the third trimester had significantly higher health anxiety scores than those in the first trimester (*p* = 0.045).

**Conclusion:**

At the time of the COVID-19 pandemic, women in the second and third trimesters of pregnancy were more worried about consequences of disease, but total health anxiety scores were significantly higher among women in the third trimester of pregnancy. Health care providers should pay more attention to the mental health of pregnant women in times of crises such as the COVID-19 pandemic.

## Background

Health anxiety is defined as the extensive worry that people experience about their health condition [1]. Health anxiety may manifest as two types: illness anxiety disorder and somatic symptom disorder, and symptoms of anxiety may vary from mild to severe with clinical signs. Pregnancy-specific anxiety is anxiety disorder, that a woman may experience when she conceives, because of either immediate somatic changes or illness anxiety disorder [2].

Stress and anxiety during pregnancy are associated with poor outcomes for women including preeclampsia, low birth weight, depression and more nausea and vomiting [3]. Women with anxiety during pregnancy may experience symptoms such as worry, stress, difficulty staying calm, sleep disturbances and negative thoughts that may prevent good sleep [4]. Anxiety during pregnancy often co-exists with depression [5]. Disorders such as perinatal depression may be associated with poorer outcomes of pregnancy [6].

COVID-19 caused by SARS-COV-2 infection in humans and has affected more than 50 million persons around the world [7]. Worry during the COVID-19 pandemic among pregnant women may lead them to refrain from attending clinics for regular prenatal care or to undergo unnecessary Cesarean-section because of fear of mother-to-neonate disease transmission [8]. Moyer et al. (2020) evaluated levels of stress and anxiety among pregnant women before and after COVID-19 pandemic. Their results showed that levels of stress and anxiety of pregnant women increased during this pandemic and that most of this anxiety is independent from pregnancy-specific anxiety [9]. Additionally, a systematic review by Hassami et al. (2020) showed that depression and anxiety scores were higher among pregnant and postpartum women during the COVID-19 pandemic [10].

Studies have suggested that viral respiratory diseases may cause pneumonia in pregnant women, which may lead to premature rupture of membranes, preterm labor, intrauterine fetal demise, intrauterine growth retardation and even neonatal death [11].

Limited evidence from pregnant women affected by COVID-19 in China and the USA reveal that more than 95% of these women delivered their babies by Caesarean section, as the popularly held belief is that the maternal respiratory disease will be worsen with normal vaginal delivery (the rate of Caesarean section in the USA and China was 32 and 54.5% respectively before the pandemic) [12–14].

COVID-19 is a novel disease and little is known about its characteristics. Iran is facing its third wave of COVID-19 with around 500 deaths per day [15]. This study was designed to investigate health anxiety among pregnant women in different trimesters in Iran.

## Methods

This was a cross-sectional study in which 300 pregnant women were recruited. The design of this study was approved by the Ethics Committee of Ahvaz Jundishapur University of Medical Sciences (Ref No: IR.AJUMS.REC.1399.006). This study started on 20 March 2020 and completed on 10 April 2020. Oral and written informed consent was obtained from each participant. Literate pregnant women in any trimester of pregnancy were eligible for inclusion in this study. Women who had experienced stressful events in the past 6 months, those with a positive test for COVID-19 and those with known mental disorders were excluded from the study.

### Sample size

The sample size was calculated using the following formula [16]:

0.6$$ 0.6 $$ $$ \alpha =0.05,s=4.48,d=0.15\times \mathrm{s}\approx $$

$$ n>\frac{{\left({z}_{1-\raisebox{1ex}{$\alpha $}\!\left/ \!\raisebox{-1ex}{$2$}\right.}\right)}^2\times {s}^2}{d^2}=\frac{(1.96)^2\times {(4.48)}^2}{(0.6)^2}\approx 214 $$

$$ 20\%\mathrm{non}-\mathrm{responding}\approx 43 $$

$$ {n}^{\ast }>257 $$

A total number of 300 pregnant women were recruited.

### Measurements

A demographic questionnaire and the Health Anxiety Questionnaire were used to collect data. The demographic questionnaire included questions about age, parity, gravidity, number of children, economic situation, the women’s and their partners’ occupation, and the trimester of pregnancy.

The Health Anxiety questionnaire [17] consists of 18 questions about participants’ worry during the COVID-19 pandemic in Iran. Each question has four response categories ranging from zero (I am not worried about my health) to three (I spend most of my time worrying about my health). The maximum total score of this questionnaire is 54. There are three sub-scales for this questionnaire. The first is related to worry about getting sick, which is reflected in questions 5, 6, 8, 9, 11 and 12. Worry about consequences of disease is the second subscale involving questions 13, 15, 16, 17 and 18, and the third subscale deals with general health concerns, as reflected in questions 1–4, 7, 10 and 14. A total score < 27 is defined as low health anxiety, scores of 27–34 are defined as moderate health anxiety, and scores of more than 35 are defined as high health anxiety. The validity and reliability of the Persian version of Health Anxiety Questionnaire had already been assessed and approved in Iran [18]. We also included a question asking women if they thought the COVID-19 pandemic had increased their feeling of anxiety during pregnancy.

The phone numbers of pregnant women were obtained from 20 public health centers in Ahvaz. Both questionnaires were sent to those eligible pregnant women who agreed to participate via social media (WhatsApp or Telegram). The front page of the questionnaires was the written informed consent, and participants were requested to sign this form before responding to the questionnaires. The completed questionnaires were sent back to one of the researchers via the same social media.

### Statistics

All data were entered SPSS version 22. The normal distribution of continuous data was assessed using the Shapiro-Wilk test. The ANOVA test was used for comparing the data across three groups (three trimesters) and the chi-square test was used for comparing categorical data. Multiple linear regression models were used for assessing the relationship of different trimesters and health anxiety, controlling for the effects of history of infertility and results of anomaly screening. *P* < 0.05 was considered statistically significant.

## Results

**Fig. 1:**
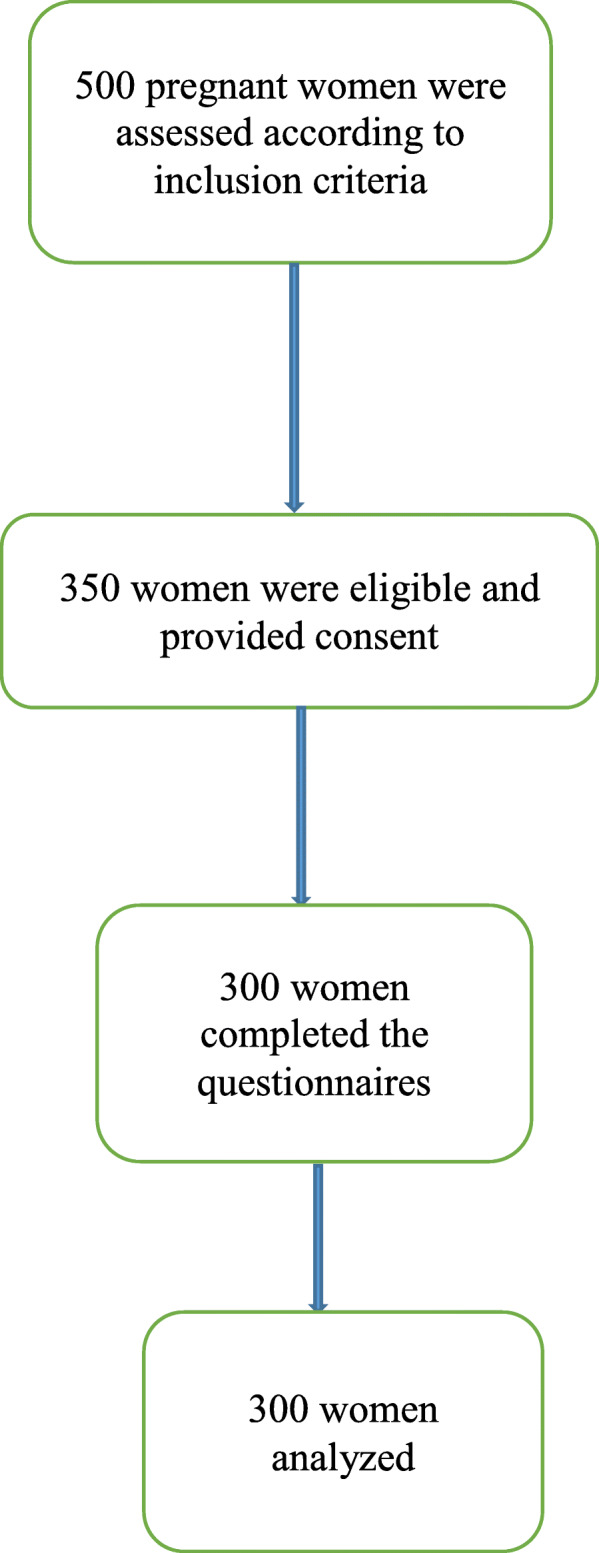
Flow-diagram of recruitment of participants in the study

Based on inclusion and exclusion criteria, we assessed 500 women of whom 350 were eligible and provided consent. A total of 300 participants returned the completed questionnaires (*n* = 100 women in each trimester) and were included in the analysis (Fig. 1). Table 1 presents the demographic and obstetric characteristics of participants in different trimesters of pregnancy. The mean (SD) age of women was 25.8 ± 5.1, 27.2 ± 5.7 and 26.5 ± 4.5 in the first, second and third trimesters, respectively (*p* > 0.05). Women did not show any significant difference regarding occupation, education, economic situation and their spouses’ level of education across the three trimesters of pregnancy.

**Table 1:** Socio-demographic and maternity characteristics of participants by trimester of pregnancy

Variables	First trimester *n* = 100	Second trimester n = 100	Third trimester n = 100	All trimesters *N* = 300	*P* value
Mean ± SD					
Age (y)	25.8 ± 5.1	27.2 ± 5.7	26.5 ± 4.5	26.54 ± 5.17	0.17
Gravida	1.72 ± 1	1.83 ± 1.1	1.91 ± 1.1	1.82 ± 1.09	0.47
Para	0.63 ± 0.9	0.69 ± 0.92	0.74 ± 0.94	0.69 ± 0.92	0.70
Living child	0.59 ± 0.86	0.66 ± 0.92	0.73 ± 0.93	0.66 ± 0.90	0.55
**Occupation**	N (%)				
Housewife	80(80)	80(80)	86 (86)	246 (82)	0.32
Employee	20 (20)	20 (20)	14 (14)	54 (18)	
**Education**					
Primary	5 (5)	12 (12)	7 (7)	24 (8)	0.65
Secondary	12 (12)	9 (9)	8 (8)	29 (9.7)	
Diploma	44 (44)	42 (42)	44 (44)	130 (43.3)	
University	39 (39)	37 (37)	41 (41)	117 (39)	
**Economic situation**					
Weak	21 (21)	21 (21)	17 (17)	59 (19.7)	0.67
Moderate	72 (72)	67 (67)	71 (71)	210 (70)	
Good	7 (7)	12 (12)	12 (12)	31 (10.3)	
**Education of spouse**					
Illiterate	1 (1)	7 (7)	1 (1)	9 (3)	0.11
Primary	6 (6)	4 (4)	6 (6)	16 (5.3)	
Secondary	15 (15)	16 (16)	11 (11)	42 (14)	
Diploma	44 (44)	31 (31)	42 (42)	117 (39)	
University	34 (34)	42 (42)	40 (40)	116 (38.6)	
**Results of anomaly screening**					
Normal	66 (66)	95 (95)	93 (93)	254 (84.7)	< 0 .0001
Suspicious or did not perform	34 (34)	5 (5)	7 (7)	46 (15.3)	
**History of infertility**	8 (8)	4 (4)	15 (15)	27 (9)	0.023
**Problems in the current pregnancy**					
Yes	25 (25)	19 (19)	35 (35)	79 (26.3)	0.097
No	75 (75)	81 (81)	65 (65)	221 (73.6)	
**If COVID-19 pandemic increased women’s anxiety**					
No	34 (34)	23 (23)	22 (22)	79 (26.3)	0.102
Yes	66 (66)	77 (77)	78 (78)	221 (73.6)	

As evident from Table 1, 95% of women in the second trimester of pregnancy had a normal anomaly screening, while 32% of women in the first trimester did not perform these tests or had test with suspicious results. Women in three trimesters showed a significant difference regarding anomaly screening (*p* < 0.0001). The three groups also showed a significant difference regarding history of infertility (*p* = 0.02). As with concerns about their pregnancy, 25, 19 and 35% of women in their first, second and third trimester, respectively, reported concerns during their pregnancy. These concerns included bleeding in the first trimester, nausea and vomiting, gestational diabetes, and hypertension (*p* = 0.097). Also, 73.6% of women reported that the COVID-19 pandemic had increased their anxiety; most of these women were in the third trimester of pregnancy (78%).

Table 2 shows levels of anxiety among women by pregnancy trimester. Women in the third trimester were more worried about getting sick, the consequences of the disease, and had more concerns about the disease. Mean (SD) anxiety scores were 22.3 ± 9.5, 24.6 ± 9.3 and 25.4 ± 10.6 in the first, second and third trimester of pregnancy, respectively. 9, 13 and 21% of women had severe anxiety (scores ≥35) in the first, second and third trimester of pregnancy, respectively.

**Table 2:** Mean (SD) anxiety scores by trimester of pregnancy

Variable	First trimester n = 100	Second trimester n = 100	Third trimester n = 100	Total N = 300
**Health anxiety**	Mean ± SD			
More worried about getting sick	7.8 ± 3.6	8.5 ± 3.5	8.7 ± 4.2	8.39 ± 3.82
More worried about consequences of the disease	5.5 ± 3.2	6.9 ± 3.1	6.8 ± 3.4	6.43 ± 3.28
Reported more concerns about disease	8.9 ± 4.1	9.2 ± 4.07	9.7 ± 4.4	9.32 ± 4.20
Total score anxiety	22.3 ± 9.5	24.6 ± 9.3	25.4 ± 10.6	24.15 ± 9.93
**Total score of health anxiety category**				
N (%)				
< 27	60 (60)	48 (48)	40 (40)	148 (49.3)
95% CI	(50.0–69.0)	(38.0–58.0)	(31.0–49.0)	(44.0–55.0)
27–34	31 (31)	39 (39)	39 (39)	109 (36.3)
95% CI	(22.0–39.0)	(30.0–48.0)	(29.0–48.0)	(30.7–41.7)
≥ 35	9 (9)	13 (13)	21 (21)	43 (14.3)
95% CI	(4.0–15.0)	(6.0–20.0)	(13.0–29.0)	(10.7–18.3)

Using multiple linear regression, significant association was found between pregnancy trimester and “being worried about the consequences of disease” after controlling for the effects of history of infertility and results of anomaly screening. Pregnant women in the second and third trimesters had significantly higher scores of “being worried about consequences of the disease”, compared to those in the first trimester (*p* = 0.010 and *p* = 0.009; respectively). Also, pregnant women in the third trimester reported significantly higher health anxiety scores than women in the first trimester. Pregnant women in the third trimester had significantly higher scores of “total health anxiety”, in comparison with those in the first trimester (*p* = 0.045). However, no significant difference was found in terms of “total health anxiety” between the second and the first trimester (Table 3).

**Table 3:** Results of multiple linear regression analyses to determine parameters most predictive interested outcomes

Outcomes	Worry to get sick			Being worry about consequences of disease			Concerns about disease			Total health anxiety		
Parameter	Beta	95% CI for Beta	P	Beta	95% CI for Beta	P	Beta	95% CI for Beta	P	Beta	95% CI for Beta	P
**Results of anomaly screening.**												
Unknown or suspicious	Ref	–	–	Ref	–	–	Ref	–	–	Ref	–	–
Normal	0.04	(−1.26,1.35)	0.950	0.33	(−0.77,1.45)	0.548	0.05	(−1.39,1.49)	0.941	0.43	(−2.95,3.82)	0.801
**History of infertility**												
Negative	Ref	–	–	Ref	–	–	Ref	–	–	Ref	–	–
Positive	−0.75	(−2.3,0.80)	0.341	−0.46	(−1.78,0.85)	0.488	0.23	(−1.47,1.95)	0.783	−0.97	(−5.00,3.04)	0.633
**Trimester**						0.011						
First trimester	Ref	–	–	Ref	–	–	Ref	–	–	Ref	–	–
Second trimester	0.62	(−0.50,1.76)	0.277	1.26	(0.30,2.22)	0.010	0.25	(−0.99,1.50)	0.690	2.14	(−0.79,5.08)	0.152
Third trimester	0.97	(−0.15,2.09)	0.089	1.27	(0.31,2.22)	0.009	0.73	(−0.49,1.97)	0.241	2.98	(0.07,5.88)	0.045

Note: The results from multiple linear regression models including each parameter controlling for the effects of history of infertility and results of anomalies screening

## Discussion

This study was designed to evaluate the health anxiety of pregnant women and its related factors during the COVID-19 pandemic in Iran. The results of this study show that compared with those in their first or second trimester, pregnant women in the third trimester were more worried about getting sick, the consequences of the disease, and concerns about disease. Also, total (mean?) anxiety scores were higher among women in the third trimester of pregnancy. A possible explanation for this may be women’s lack of access to their health providers in time of delivery, or they may be reluctant to go to healthcare facilities or hospitals since they consider such places as being unsafe environments during COVID-19 pandemic [19]. A qualitative study by Mizrak et al. (2020) in Turkey showed that fear of the unknown, disruption of routine prenatal care and disruption of social life because of quarantine caused anxiety in pregnant women during the COVID-19 pandemic [20]. Another study by Kahyaoglu et al. (2020) in Turkey showed that the prevalence of anxiety and depression among pregnant women during COVID-19 pandemic was 64.5 and 56.3%, respectively [21]. Our results are in line with these studies. According to Werner et al. (2020), the use of new and diverse models of prenatal care by health care providers can reduce the anxiety of pregnant women in crises such as COVID-19 pandemic [22].

The results of the present study showed that women in the third trimester of pregnancy were more prone to be worried and also had significantly more health anxiety compared to women in the second and the first trimesters of pregnancy. Other studies showed that pregnant women are more worried about different problems in the second and third trimesters of pregnancy [23]. In the present study we controlled some confounding factors such as anomaly screening tests. According to the national guidelines in Iran, women should undergo anomaly screening tests including nuchal translucency, and PAPP-A between the 11th and 13th week of gestation. If there is any abnormality in these tests, then women are encouraged to undergo further tests around the 15th week of gestation. We found that 34% of women in the first trimester did not perform any anomaly test or had test results which suggested were abnormal. This may cause undue anxiety in pregnant women in addition to COVID-19-related anxiety.

A study by Corbett et al. (2020) [24] showed that most pregnant women (83.1%) did not worry about their health status before the pandemic of COVID-19, but during the pandemic, 50.7% were worried about their health status most of the time. Concerns of pregnant women may be related to the fact that they do not have access to their relatives if they needed, and many pregnant women may have concerns about lack of family and social support due to distancing measures [25]. In the first days of COVID-19 pandemic in Iran, one hospital in Ahvaz, where a large number of women receive intrapartum care, was designated as the center for patients with COVID-19 infection. Although another hospital was later redeveloped to care for pregnant women, the change in location of care may have contributed to women’s symptoms of anxiety.

### Limitations of the study

Women were recruited non-randomly in this study, which may limit the generalizability of the study. Also, we may not reach a representative sample, because we recruited only women who had social media. Furthermore, a past history of depression or anxiety, and levels of social support were not assessed in the present study and all of these have the potential to contribute to health anxiety.

## Conclusion

At the time of COVID-19 pandemic, women in the second and third trimester of pregnancy were more worried about the consequences of disease, but the mean scores of health anxiety was significantly higher in the third trimester of pregnancy. Healthcare providers should pay more attention to the mental health of pregnant women and provide more psychological support to them in times of crises such as the COVID-19 pandemic. Also, further studies about specific causes of women’s anxiety and identifying supportive mechanisms during COVID-19 pandemic are needed.

## Data Availability

Data will be available upon the request from corresponding author.

## Abbreviations

ANOVA: Analysis of Variance
COVID-19: Corona Virus Disease 2019
WHO: World Health Organization
SARS: Severe acute respiratory syndrome
